# Hyperosmotic stress enhances cytokine production and decreases phagocytosis *in vitro*

**DOI:** 10.1186/cc6989

**Published:** 2008-08-18

**Authors:** Natalie M Otto, Ralf Schindler, Andreas Lun, Olaf Boenisch, Ulrich Frei, Michael Oppert

**Affiliations:** 1 Department of Nephrology and Medical Intensive Care, Charité Universitätsmedizin Berlin, Humboldt University, Augustenburger Platz 1, 13353 Berlin, Germany; 2 Institute of Clinical Chemistry, Charité Universitätsmedizin Berlin, Humboldt University, Augustenburger Platz 1, 13353 Berlin, Germany

## Abstract

**Introduction:**

Hyperglycemia is associated with negative outcomes in various settings of critical illness; infectious complications, especially, seem to be increased. On the other hand, intensive insulin therapy (IIT) has been shown to improve outcome in clinical trials. Whether normoglycemia itself or the application of insulin is responsible for the observed findings is unknown. We therefore tested the effect of glucose and insulin on various immune functions *in vitro*.

**Methods:**

Human peripheral blood mononuclear cells (PBMCs) were incubated *ex vivo *with low doses of lipopolysaccharide (LPS). PBMCs were incubated with various osmotic agents, insulin, or a combination of both. Interleukin (IL)-6 and IL-1 cytokine response was measured by enzyme-linked immunosorbent assay. In addition, we investigated the effects of glucose on phagocytosis and oxidative burst in human granulocytes.

**Results:**

Increasing concentrations of both glucose and mannitol significantly enhanced LPS-induced cytokine production. Insulin alone did not alter cytokine production and had only a minor influence in combination with glucose. Phagocytosis and oxidative burst were significantly reduced with increasing concentrations of glucose and mannitol.

**Conclusion:**

Hyperglycemia may lead to inflammation by enhancing cytokine production via the direct effects of hyperosmotic stress. Impaired phagocytosis and oxidative burst under hyperglycemia may weaken defense mechanisms of the host. Our *in vitro *findings may help to explain the beneficial effects of IIT not only in diabetic but also in critically ill patients.

## Introduction

Hyperglycemia is common in acutely ill patients and may be attributed to the release of stress hormones, peripheral insulin resistance, and certain drugs. Hyperglycemia is an independent risk factor for increased morbidity and mortality in critically ill patients with a variety of conditions. After myocardial infarction, hyperglycemia is associated with an increased risk of in-hospital mortality in patients with or without diabetes [1,2]. After cardiac surgery, hyperglycemia has been associated with increased postoperative complications [3]. A randomized and prospective trial among 1,548 surgical intensive care unit (ICU) patients receiving mechanical ventilation demonstrated that intensive insulin therapy (IIT) reduced mortality from 20.2% to 10.6% in patients who remained in the ICU for more than 5 days [4]. These results were subsequently partially confirmed in patients admitted to a medical ICU [5].

The mechanisms underlying the detrimental effects of hyperglycemia and the beneficial effects of IIT are not fully understood but may be related to alterations in immune functions and inflammation [6]. Hyperglycemia enhances plasma levels of interleukin (IL)-6 and tumor necrosis factor-alpha (TNF-α) in normal healthy volunteers [7,8]. Hyperglycemia after myocardial infarction is associated with higher levels of C-reactive protein (CRP) and IL-18 [9], and in the abovementioned study [4] IIT decreased CRP levels significantly [10]. In a rabbit model, strict glycemic control with insulin improved innate immune function such as phagocytosis and prevented excessive inflammation [11]. After endotoxin injection in pigs, insulin treatment reduced the cytokine content of various organs [12].

These *in vivo *studies indicate an important relationship between hyperglycemia and immune cell function. However, the precise cellular and molecular mechanisms of hyperglycemia and insulin therapy have yet to be fully characterized. In living animals, multiple interacting mechanisms are often difficult to separate. In particular, the protective effect of insulin therapy may be due to insulin alone, the lowering of glucose, its endocrine effects, or a combination thereof. To address this question, we examined the effects of various hyperosmotic substances (glucose and mannitol), as well as insulin, on cytokine production in human peripheral blood mononuclear cells (PBMCs) *in vitro*. In addition, we investigated the effects of these hyperosmotic substances (glucose and mannitol) on phagocytic and oxidative burst activity in human granulocytes.

## Materials and methods

This study was approved by the Ethical Committee on Human Research of Charité University Hospital (Berlin, Germany) and was performed in accordance with the Declaration of Helsinki (1964). Informed consent was obtained from all volunteers.

### Preparation of peripheral blood mononuclear cells

Blood was taken from healthy volunteers (age range 24 to 46 years) without recent infectious or inflammatory conditions. PBMCs were separated from whole blood by centrifugation through Ficoll solution (Ficoll-Paque TM Plus; GE Healthcare, Bio-Sciences AB, Uppsala, Sweden). PBMCs were washed twice with normal saline, resuspended at 5 × 10^6^/mL in serum-free culture medium (RPMI 1640; PAA Laboratories GmbH, Pasching, Austria) containing glucose (100 mg/dL), supplemented with L-glutamine, penicillin, and streptomycin (Biochrom AG Seromed, Berlin, Germany). PBMCs were preincubated in 12-well plates (Nunc, Roskilde, Denmark) with different concentrations of glucose (250, 500, and 1,000 mg/dL), mannitol (500 and 1,000 mg/dL), or human insulin (10, 100, and 1,000 IU) for 3 hours at 37°C in a humidified atmosphere containing 5% CO_2_. After preincubation, cells were stimulated with 0.5 ng/mL lipopolysaccharide (LPS) (*Escherichia coli *055:B5, Sigma-Aldrich number L6529; Sigma-Aldrich, Munich, Germany) and subsequently incubated for 24 hours. Samples were then stored at -80°C until performance of the assay.

### Cytokine assay

IL-6 and IL-1β levels were quantified by enzyme-linked immunosorbent assay (ELISA) after two freeze-thaw cycles. Plates (96-well; Maxisorp, Nunc) were coated overnight with the primary antibody (50 μL/well; R&D Systems, Wiesbaden-Nordenstadt, Germany) in a coating buffer (0.2 M NaHCO_3_/Na_2_CO_3_, pH 10.5). Wells were then blocked with casein (0.2%; Sigma-Aldrich) in phosphate-buffered saline (PBS) for 1 hour, after which sample and standard probes were added to each well (50 μL/well) and incubated overnight. After three to five washings, appropriately diluted biotinylated secondary antibody (R&D Systems) was added to each well (50 μL/well) and incubated for 1 hour. Plates were then incubated with peroxidase-streptavidin-biotin complexes (50 μL/well; Amersham, now part of GE Healthcare, Braunschweig, Germany) for 1 hour and then developed with TMB (240 μg/mL 3,3',5,5' tetramethylbenzidine; Fluka Chemicals, Buchs, Switzerland) in Gallati buffer (42 μg/mL citric acid, pH 3.95/0.01% H_2_O_2_). All dilutions were made in PBS containing 0.05% Tween (Sigma-Aldrich), and wells were washed with PBS-Tween after each incubation step. Cytokine levels in each sample were determined by measuring the optical density at 450 and 630 nm on an ELISA plate reader (Dynastar MR5000, Dynatech, Chantilly, VA, USA). Samples were measured in duplicate in at least two dilutions until values conformed to the linear part of the standard curve. For IL-6, the sensitivity of the cytokine assay varied between 5 and 10 pg/mL.

### Phagocytosis assay

Phagocytosis was quantified by measuring the overall percentage of monocytes or granulocytes showing ingestion of bacteria per cell and individual cellular phagocytic activity was then analyzed. Heparinized whole-blood samples were incubated with fluorescein isothiocyanate (FITC)-labeled *E. coli *bacteria (1 × 10^5 ^bacteria per microliter of incubation medium) at body temperature (37°C). As a control, one probe was left on ice. Samples were then washed twice with PBS-Tween, after which erythrocytes were lysed by adding prewarmed lysis buffer. DNA staining solution was added prior to flow cytometric analysis, excluding artifacts from bacteria or aggregating cells. Granulocyte cell populations were gated using a forward (FSC) and side (SSC) scatter to assess mean FITC fluorescence activity. Granulocyte metabolism and production of reactive oxygen species (ROS) led to morphological changes that could be detected in the FSC and SSC. With the CD14 surface marker, an additional assay of location and cell population allocation was performed.

One sample was placed on ice and bathed in ice-cold quenching solution prior to erythocyte lysis, in order to prevent active phagocytosis. Fluorescence activity of this frozen probe provided a measure of the nonspecific binding of *E. coli *with the granulocytes. Therefore, differential fluorescence activity of the lysed population provided a quantification of active phagocytosis. Significant active phagocytosis was considered to have occurred in samples in which mean fluorescence activity of the heated probe exceeded 300% of the activity measured in the inoculated probe left on ice [13].

### Oxidative burst activity assay

Heparinized whole-blood samples were incubated with various stimuli at 37°C. Unlabelled opsonized *E. coli *bacteria were used as a particulate stimulus, the protein C ligand phorbol 12-myristate 13-acetate (PMA) was used as a high stimulus, and the chemotactic peptide *N*-formyl-MetLeuPhe (fMLP) subsequently was used as a low physiological stimulus. Dihydrorhodamine 123 served as a fluorogenic substrate. The negative control was a sample without stimulus. Whole-blood samples were then lysed, washed, and stained to exclude aggregation artifacts of bacteria or cells. The percentage of cells having produced reactive oxygen radicals was then analyzed as well as their mean fluorescence intensity (enzymatic activity).

### Flow cytometric analysis

Cells were analyzed by flow cytometry using the blue-green excitation light (488 nm argon-ion laser, BD FACScan with CellQuest software; BD Biosciences, San Jose, CA, USA). During data acquisition, a 'live' gate was set in the red fluorescence histogram on those events, which had at least the same DNA content as a human diploid cell. Alternatively, bacteria could be excluded by using the fluorescence triggering in the FL2 or FL3 channel. Leukocytes (10,000 to 15,000 per sample) were collected. The percentage of cells having performed phagocytosis (granulocytes and monocytes) was analyzed as well as their mean fluorescent intensity (number of ingested bacteria) and thus the percentage of cells having produced reactive oxygen metabolites (recruitment) was analyzed as well as their mean fluorescence intensity (amount of cleaved substrate, activity). For that purpose, the relevant leukocyte cluster was gated in the software (lin FSC versus lin SSC) and its green fluorescence histogram (fluorescence-1, FL1) was analyzed. The control sample was set as a marker for FL1 so that fewer than 1% of the events were positive. The percentage of phagocytosing cells in the test sample was then determined by counting the number of events above the marker position. The mean fluorescence correlates with the number of bacteria per individual leukocyte for the phagocytosis and oxidation quantity per individual leukocyte for the oxidative burst activity assay. The test kit for the quantification of the oxidative burst activity and the phagocytic activity of monocytes and granulocytes in heparinized whole blood was obtained from ORPEGEN Pharma (Heidelberg, Germany).

### Statistical analysis

Values were tested for normal distribution (Shapiro-Wilk test and D'Agostino and Pearson omnibus normality test) and were expressed as mean ± standard error of the mean as indicated. Groups were compared using repeated measures one-way analysis of variance with *post hoc *testing by Bonferroni. A *P *value of 0.05 or less was considered to be significant. Data were analyzed using Prism 5 (GraphPad Software Inc., San Diego, CA, USA).

## Results

### Effect of glucose on cytokine production

**Figure 1:**
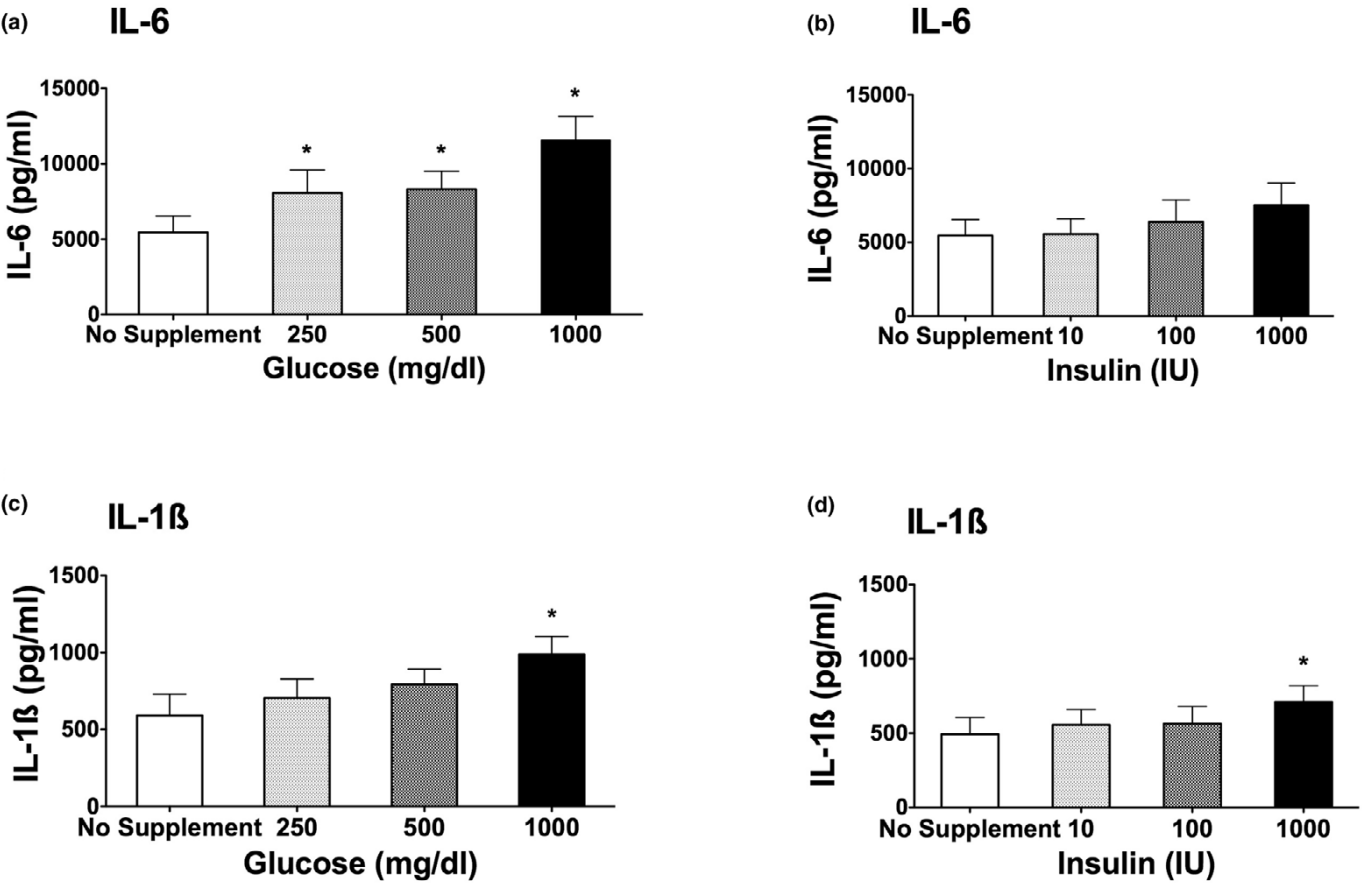
Interleukin (IL)-6 **(a, b) **and IL-1β **(c, d) **cytokine release after lipopolysaccharide (LPS) stimulation. Samples were incubated with no supplement addition, increasing concentrations of glucose (250, 500, and 1,000 mg/dL) **(a, c)**, and increasing concentrations of insulin (10 and 100 IU) **(b, d)**. Stimulation occurred after a 3-hour preincubation with 0.5 ng/mL LPS. Cytokine release was measured by enzyme-linked immunosorbent assay after another 24-hour incubation period (n = 11; mean ± standard error of the mean; *P < 0.05 versus no supplement addition).

Exposure of PBMCs to hyperglycemic medium enhanced IL-1β and IL-6 production in a concentration-dependent manner when compared with no supplement addition (iso-osmolar) medium. Figures 1a and 1c demonstrate that, under increasing concentrations of glucose, the LPS-stimulated production of IL-6 and IL-1β is significantly enhanced (*P *< 0.05). As little as 250 mg/dL glucose led to a significant elevation of IL-6 levels compared with no supplement addition (Figure 1a; *P *< 0.05). A dose-dependent effect could be demonstrated. The production of IL-1β was significantly enhanced only at a supplementation of 1,000 mg/dL glucose to the medium (Figure 1b; *P *< 0.05). Without LPS stimulation, increasing concentrations of glucose (250, 500, and 1,000 mg/dL) did not cause an increase in IL-1β and IL-6 cytokine release compared with samples stimulated with LPS. For IL-1β, 0 mg/dL glucose = 7.7 pg/mL; 250 mg/dL glucose = 7.3 pg/mL; 500 mg/dL glucose = 5.9 pg/mL; and 1,000 mg/dL glucose = 19.4 pg/mL. For IL-6, 0 mg/dL glucose = 23.4 pg/mL; 250 mg/dL glucose = 30.0 pg/mL; 500 mg/dL glucose = 53.1 pg/mL; and 1,000 pg/mL glucose = 53.2 pg/mL.

### Effect of insulin on cytokine production

Insulin alone at increasing concentrations failed to alter the LPS-stimulated IL-6 production (Figure 1b). Only in the case of IL-1β, the highest insulin level (1,000 IU/mL) led to a small but significant increase in cytokine production (Figure 1d).

### Combination of glucose and insulin on cytokine release

**Figure 2:**
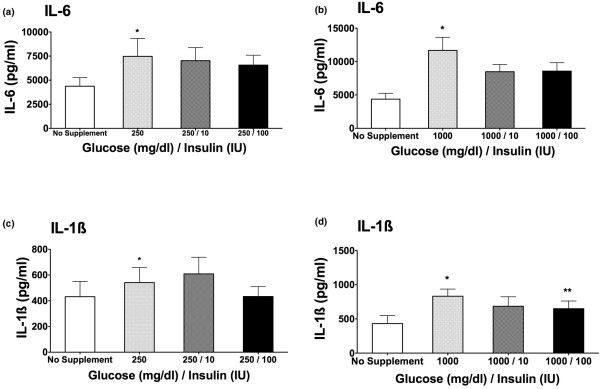
Interleukin (IL)-6 **(a, b) **and IL-1β **(c, d) **cytokine production after stimulation with 0.5 ng/mL lipopolysaccharide. Samples were preincubated for 3 hours with no supplement addition, 1,000 mg/dL glucose, or a combination of glucose (1,000 mg/dL) and insulin (10 and 100 IU). Cytokine concentrations were determined by enzyme-linked immunosorbent assay after a 24-hour incubation period (n = 11; mean ± standard error of the mean; *P < 0.05 versus no supplement addition; **P < 0.05 versus 1,000 mg/dL glucose).

To determine whether the hyperglycemic effect on cytokine release seen in Figures 1a and 1c could be reversed by the addition of insulin to the samples incubated with glucose, we next tested the effect of a combination of glucose and insulin on IL-6 and IL-1β cytokine production. The addition of insulin to a high-glucose medium partially reversed the augmentation of cytokine production mediated by glucose. The difference was statistically significant only at the highest concentration of both insulin (100 IU) and glucose (1,000 mg/dL) when measuring IL-1β cytokine release (Figure 2d; *P *< 0.05).

### Effect of hyperosmolarity on cytokine production

**Figure 3:**
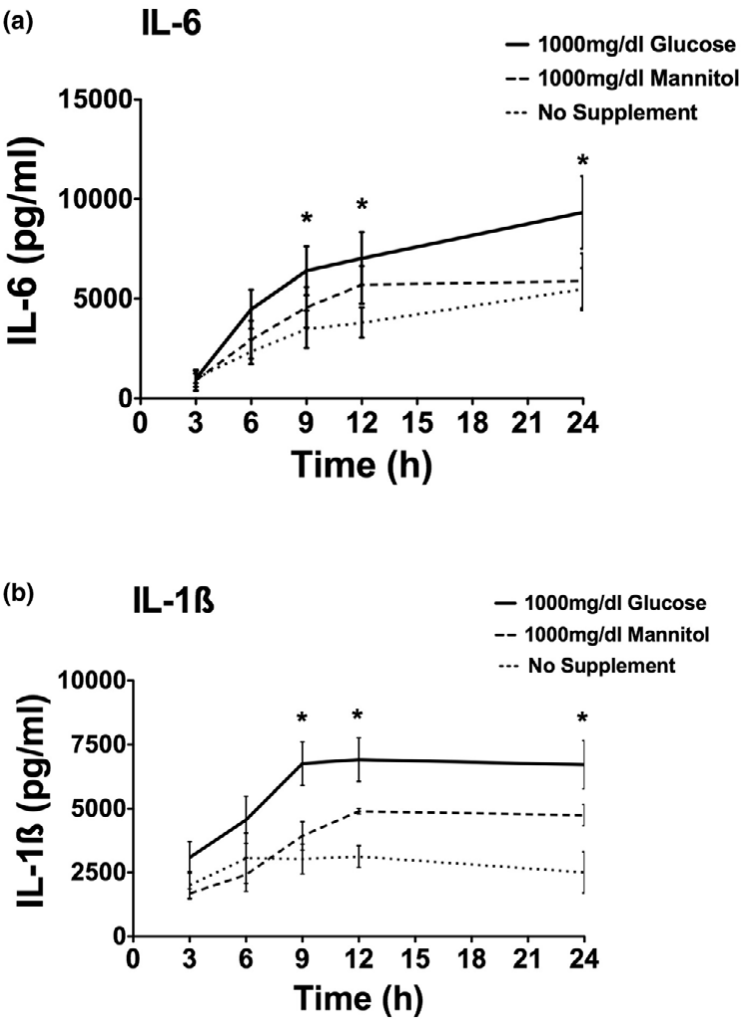
Interleukin (IL)-6 **(a) **and IL-1β **(b) **cytokine production over a time period of 24 hours after stimulation with 0.5 ng/mL lipopolysaccharide. Samples were preincubated for 3 hours under no supplement addition and equal concentrations of glucose or mannitol (1,000 mg/dL). After 3, 6, 9, 12, and 24 hours of incubation, cytokine production was measured via enzyme-linked immunosorbent assay (n = 4 to 5; mean ± standard error of the mean; *P < 0.05 versus no supplement addition).

Using a different osmotic agent, we next investigated the influence of mannitol on cytokine production. PBMCs were preincubated with glucose (1,000 mg/dL) or mannitol (1,000 mg/dL). Figure 3 shows the IL-1β and IL-6 cytokine release over a 24-hour time period. The addition of 1,000 mg/dL glucose to the medium led to a significant and prolonged increase in stimulated cytokine production compared with no glucose (*P *< 0.05). The addition of mannitol (1,000 mg/dL) to the medium also enhanced cytokine response; however, the augmentation by mannitol was not as prominent compared with glucose and did not reach statistical significance.

### Effect of glucose and mannitol (hyperosmolarity) on oxidative burst activity

**Figure 4:**
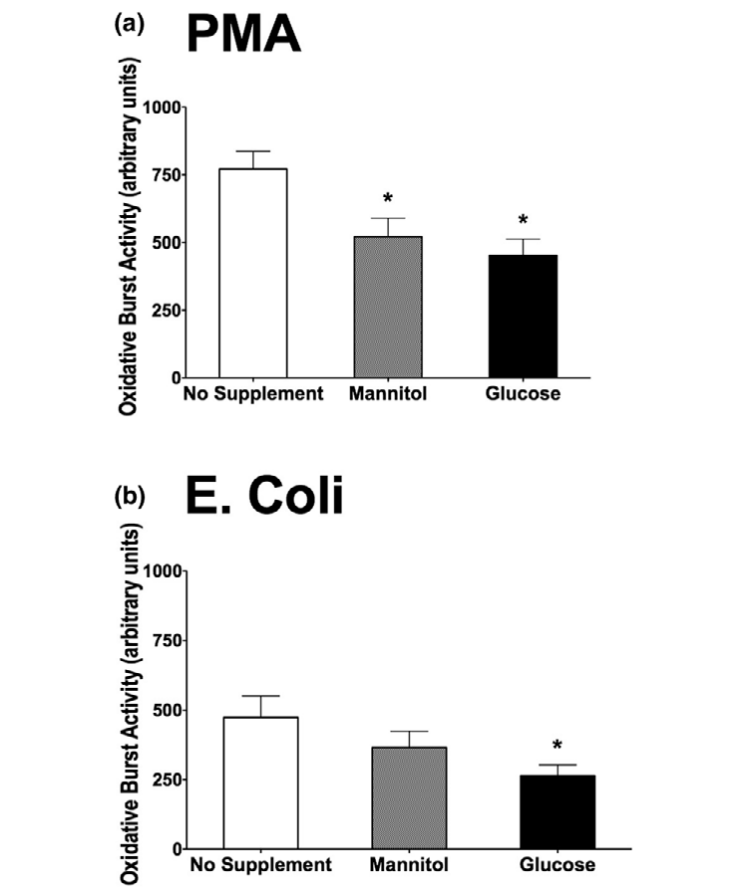
Oxidative burst activity with phorbol 12-myristate 13-acetate (PMA) **(a) **and *Escherichia coli ***(b) **as stimuli. Samples were incubated for 3 hours with no supplement addition, mannitol (500 mg/dL), and glucose (500 mg/dL). Oxidative burst activity was determined via flow cytometry (n = 10; mean ± standard error of the mean; *P < 0.05 versus no supplement addition).

We also examined the oxidative burst and phagocytic activity in whole blood under the influence of hyperglycemic and hyperosmotic conditions. The granulocytic oxidative burst activity using PMA (Figure 4a) or *E. coli *(Figure 4b) as stimuli was significantly reduced under hyperglycemic conditions. Adding the equal amount of mannitol (500 mg/dL) to the samples also led to a reduction of oxidative burst activity that was significant when using PMA, but not *E. coli*, as stimulus (Figure 4).

### Effect of glucose and mannitol (hyperosmolartiy) on phagocytic activity

**Figure 5:**
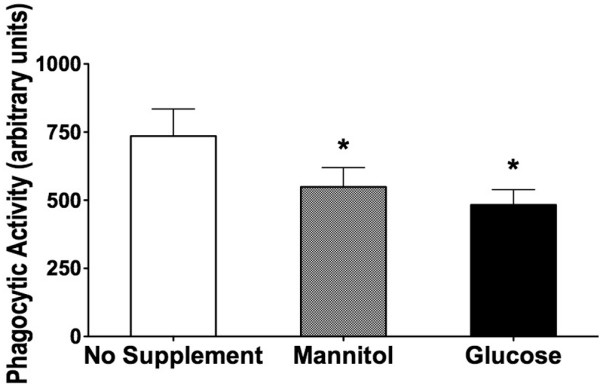
Granulocytic phagocytic activity. Samples were incubated for 3 hours with no supplement addition, mannitol (500 mg/dL), and glucose (500 mg/dL) (n = 10; mean ± standard error of the mean; *P < 0.05 versus no supplement addition).

The addition of glucose (500 mg/dL) to the samples resulted in a significantly reduced rate of phagocytosis in whole blood compared with no supplement addition (Figure 5; *P *< 0.05). Similar to the effect on oxidative burst activity, the addition of mannitol (500 mg/dL) to the medium also led to a significant decrease in phagocytic activity (Figure 5).

## Discussion

Our results demonstrate that high concentrations of glucose enhance LPS-induced cytokine production in fresh human PBMCs. In addition, glucose decreases phagocytosis and oxidative burst in whole blood. The effects of glucose appear to be mediated by hyperosmotic stress since similar, though weaker, effects could be observed using mannitol. Our findings are in line with observations in animal models of critical illness [11,12]. In a porcine model, stress hyperglycemia led to a significantly enhanced inflammation as shown by elevated TNF-α and IL-6 levels [12]. This effect could be partly reversed by the addition of insulin. Similarly, burn injury in rabbits [11] leads to stress hyperglycemia and elevation of inflammatory markers. The addition of insulin to maintain normoglycemia resulted in reduced inflammation 3 days after the injury compared with rabbits under hyperglycemia. Experimental hyperglycemia was also shown to elevate circulating cytokine levels in healthy volunteers as well as in diabetic subjects [7]. Interestingly, diabetic patients had significantly higher cytokine levels even before the glucose challenge leading to hyperglycemia. After endotoxin stimulation, the induction of hyperglycemia led to a slight but significant increase in IL-6 in healthy volunteers, whereas TNF-α levels were unaffected [8]. The addition of insulin to reach normoglycemia, however, did not alter the cytokine response in healthy volunteers. In critically ill surgical patients, however, IIT led to a reduced level of inflammation [4]. In this prospective randomized controlled trial involving 1,548 adult patients, van den Berghe and colleagues [4] demonstrated that IIT reduced mortality during intensive care from 8.0% to 4.6%.

The greatest reduction in mortality involved deaths due to multiple-organ failure with a proven septic focus. IIT also reduced overall in-hospital mortality, bloodstream infections, and acute renal failure requiring renal replacement therapy. Although cytokine levels were not measured, the level of inflammation as expressed by the level of CRP was significantly lower in patients under IIT compared with conventional insulin therapy [4]. Furthermore, the rate of secondary infections and the rate of severe sepsis decreased. The reason why critically ill patients under IIT have fewer severe infections is not fully understood. These results, however, indicate that functions of the immune system may be altered.

Several experimental studies investigating the influence of glucose, insulin, and osmotic stress on the immune system on a cellular level have been undertaken [14-19]. Glucose was shown to induce cytokine production in cell lines [14,20]. However, cell lines may react differently from freshly isolated cells due to long and artificial culture conditions. Besides the present study, only one group investigated fresh PBMCs and, in agreement with our results, reported enhancement of IL-6 and TNF production by glucose [17]. Osmotic stress by hypertonic saline in PBMCs led to conflicting results. Whereas Shapiro and Dinarello [18] found an elevation of cytokine levels, others report of reduced cytokine production under osmotic stress [15]. The present investigation confirms the results that glucose is able to induce an enhanced cytokine response in fresh human PBMCs. Although high concentrations of glucose have been used in our study, it is important to consider that only viable cells are able to produce and release cytokines. Cytokines are not stored in intracellular compartments but rather are newly synthesized and released in response to inflammatory stimuli [21,22]. It is thus very unlikely that the observed effects are due to nonspecific toxicity.

There are several potential explanations about the possible underlying molecular mechanism of cytokine induction under increasing levels of glucose and changes in osmolarity. The protein kinase C seems to be involved in the elevated cytokine production by monocytes [14]. Furthermore, it has been suggested that other mitogen-activated protein (MAP) kinases and nuclear factor-kappa-B (NF-κB) are involved as well [23-25]. Sherry and colleagues [25] could block LPS-induced TNF-α production by inhibition of p38 MAP kinase in diabetic mice. In another study, Németh and colleagues [26] observed an increase in IL-8 production when exposing intestinal epithelial cells to hyperosmotic conditions. This effect seemed to be mediated by activation of p38 and p42/44 MAP kinase as well as an increase in NF-κB [26]. In a sideline to the present study, preliminary data of our group suggest the involvement of p38 MAP kinase.

In a clinical setting, the application of insulin seems to be important as it lowers glucose levels and therefore improves metabolic and osmotic homeostasis. A direct effect of insulin on immune function, however, has not been demonstrated before. In agreement, in the present study, we did not observe that high doses of insulin alone alter the LPS-stimulated cytokine response. The effects of insulin, even in combination with glucose, were rather small and significant in only one experiment. We therefore do not believe that insulin exerts major direct effects on cytokine response.

During hyperglycemic conditions, such as inadequately controlled diabetes, phagocytosis has been reported to be impaired. Clinical trials showed a direct correlation between metabolic control of diabetes and the phagocytic capacity of polymorphonuclear (PMN) cells [27]. These findings are supported by several animal studies: in one study, rabbits with experimental diabetes were randomly assigned to receive insulin treatment followed by a 30% burn injury. In the group receiving insulin, phagocytic activity of monocytes improved by 150%; similarly, a twofold augmentation of oxidative killing compared with controls was observed [11].

In accordance with our findings, several previous studies support the notion that not only hyperglycemia but also changes in osmolarity induce alterations in PMN cell function [19,27-29]. Impairment of phagocytic/oxidative burst activity under increasing levels of glucose and changes in osmolarity may have different explanations. Intracellular killing and phagocytosis are partly transmitted via the formation of oxygen species (ROS). Hyperglycemia was shown to inhibit ROS formation in several studies [30]. Alexiewicz and colleagues [31] linked the impaired phagocytic activity to elevated intracellular calcium [Ca^2+^]_I _levels as they observed a significant direct relation between [Ca^2+^]_I _and the degree of hyperglycemia. They also suggested a glucose-induced calcium influx as well as an increased activity of protein kinase C to be responsible for this effect. They hypothesized that the acute increase in intracellular calcium would deplete the cell's adenosine triphosphate content necessary for the conformational changes during phagocytosis [18,32]. However, whether the observed *in vitro *effects of hyperglycemia and hyperosmolarity can explain the reported clinical benefits of IIT remains unknown. A study in healthy human subjects undergoing a 4-hour hyperglycemic or hyperinsulinemic euglycemic clamp test demonstrated no significant changes in PMN cell function [33], indicating that a short acute hyperglycemia may not be sufficient to provoke changes in PMN cell function. Only a more complete understanding of innate immunity during alterations of the host's physiologic milieu will enable the correlation of the observed immunologic and metabolic alterations to the clinical outcome of the patients. Many other aspects of innate immunity, such as neutrophil apoptosis, chemotactic migration of monocytes and neutrophils, complement-mediated cell lysis, the kininogen-bradykinin system, or the role of the mast cell, still remain poorly understood regarding their functional alterations during acute hyperglycemia.

## Conclusion

Clinical trials demonstrate considerable evidence for the benefits of IIT in critically ill surgical patients. Our study focused on the *in vitro *aspects of acute hyperglycemia, hyperinsulinema, and hyperosmotic stress on the human innate immune system *in vitro*. We could demonstrate that cytokine production by fresh human PBMCs is enhanced under hyperglycemic and hyperosmotic conditions. Furthermore, an impairment of neutrophil phagocytic and oxidative burst activity by glucose was shown. Our *in vitro *findings may help to explain the beneficial effects of IIT not only in diabetic but also in critically ill patients.

## Key messages

• Increasing glucose concentrations *in vitro *enhance interleukin (IL)-6 and IL-1β cyotkine release by peripheral blood mononuclear cells (PBMCs).

• Addition of insulin alone has no effect on cytokine release.

• Hyperosmolarity is also able, to a lesser extent, to enhance cytokine release by PBMCs.

• Phagocytosis and oxidative burst are reduced under increasing glucose and mannitol concentrations.

## Abbreviations

CRP = C-reactive protein; ELISA = enzyme-linked immunosorbent assay; FITC = fluorescein isothiocyanate; FL1 = fluorescence-1; FSC = forward scatter; ICU = intensive care unit; IIT = intensive insulin therapy; IL = interleukin; LPS = lipopolysaccharide; MAP = mitogen-activated protein; NF-κB = nuclear factor-kappa-B; PBMC = peripheral blood mononuclear cell; PBS = phosphate-buffered saline; PMA = phorbol 12-myristate 13-acetate; PMN = polymorphonuclear; ROS = reactive oxygen species; SSC = side scatter; TNF-α = tumor necrosis factor-alpha.

## Competing interests

The authors declare that they have no competing interests.

## Authors' contributions

NMO helped to plan the study, performed all experiments, and helped to analyze the data and draft the manuscript. RS helped to plan the study, supervised all experiments, and helped to analyze the data and draft the manuscript. AL and OB helped to plan the study, perform some experiments, analyze the data, and revise the manuscript for important intellectual content. UF helped to plan the study, analyze the data, and revise the manuscript for important intellectual content. MO helped to plan the study, analyze the data, and draft the manuscript. All authors read and approved the final manuscript.
